# Risk Identification and Mitigation of Skin and Soft Tissue Infections in Military Training Environments

**DOI:** 10.3390/tropicalmed9120306

**Published:** 2024-12-14

**Authors:** Rebecca Suhr, Amy Peart, Brian Vesely, Michael Waller, Andrew Trudgian, Christopher Peatey, Jessica Chellappah

**Affiliations:** 1 Australian Defence Force Malaria and Infectious Disease Institute, Enoggera, QLD 4051, Australia; 2 Microbiology Department, Melbourne Pathology, Collingwood, VIC 3066, Australia; 3 Walter Reed Army Institute of Medical Science-Armed Forces Research Institute of Medical Sciences (WRAIR-AFRIMS), Bangkok 10400, Thailand; 4 School of Public Health, University of Queensland, Herston, QLD 4006, Australia; 5 QIMR Berghoefer—Queensland Medical Institute, Herston, QLD 4006, Australia

**Keywords:** cellulitis, staphylococcus, MRSA, military, hygiene, skin infection

## Abstract

Objective: Staphylococcus aureus (SA), including methicillin-resistant strains (MRSAs), is a major cause of skin and soft tissue infections (SSTIs) in military populations. This study investigated SSTI incidence and SA carriage in a military training site over 16 weeks using a prospective observational cohort design. Methods: Two training cohorts provided pre- and post-training self-collected swabs for bacterial carriage, and environmental swabs from accommodations, personal items, and training facilities. Hygiene awareness and practices were assessed through questionnaires. Bacteria were identified using culture, mass spectrometry (MALDI-TOF), and genomic sequencing. Results: Nasal carriage of SA increased from 19% to 49% by the end of training. SSTIs requiring treatment occurred in 16% of participants. Steam cleaning reduced but did not eliminate SA on personal bed linen. Additionally, 40% of participants had poor knowledge of antibacterial cleaning practices and wound management. Conclusions: Increased SA carriage was linked to human-to-human transmission in close-quarter military training environments. Implications for Public Health: Improved personal hygiene training, wound management education, and monitored cleaning protocols are essential to mitigate SSTI risks in communal military training environments.

## 1. Introduction

### 1.1. Staphylococcus and SSTIs

*Staphylococcus aureus* (SA), also called *S. aureus* or “staph”, is a bacterium that commonly colonises the human skin and nares in about 25 to 30 per cent of adults. SA can exist in this form without harming its host or causing symptoms. However, if there is a break in the skin from a wound, surgery, or intravenous access device, or if there is a suppression of a person’s immune system, SA can cause an opportunistic infection. It can infect the bloodstream, skin and soft tissues, and lower respiratory tract and can cause infections related to medical instrumentation, such as central-line-associated bloodstream infection, as well as some serious deep-seated infections such as endocarditis and osteomyelitis [1,2].

Community-acquired methicillin-resistant SA (MRSA) infections have been rising in frequency since they were first described in the 1980s [3,4,5]. They have been associated with a growing number of outbreaks and deaths in non-medical settings where individuals are in close contact, such as in contact sports, daycare facilities, military units, and prisons [6,7].

Nasal carriage of SA has long been linked with skin carriage [8] and autoinfection to skin lesions [9], with the anterior nares confirmed as the most frequent site of SA carriage [10,11,12]. Local nasal antibiotic treatment can reduce nasal SA carriage and simultaneously reduce skin carriage after 15 days [8]. There are three types of nasal carriage patterns: persistent, intermittent, and non-carriage [13,14,15]. Studies have recently shown that about 20% of individuals are persistent nasal carriers of SA and around 30% are intermittent carriers, whereas about 50% are non-carriers [16]. SA can be transmitted from both infected and colonised people (with nasal carriage) to others through skin contact or through sharing contaminated objects, such as towels or razors [17,18].

### 1.2. Infections in Military Populations

*Staphylococcal* skin and soft tissue infections (SSTIs), especially those caused by MRSA, are common among military service members [6]. Prior epidemiologic studies in high-risk groups, such as military recruits, have reported MRSA colonisation rates between 2% and 6% [11,19,20,21] and have noted a higher risk of subsequent SSTIs in MRSA-colonised recruits as compared to non-colonised recruits or those colonised with MSSA. Both land-based military settings and shipboard deployments represent a high-risk environment for the spread of virulent SA strains due to crowded conditions, shared equipment, and limited opportunities for personal hygiene that facilitate colonisation [22,23].

Whilst mortality from SSTIs in soldiers is low, these conditions comprise a large proportion of solider morbidity on combat deployments and peacekeeping missions [24]. SSTIs remain prevalent in modern combat, with the self-reported incidence of SSTIs amongst surveyed deployed US military members in 2011 at 5% [25]. High rates of multi-drug-resistant organisms have been observed in recent conflicts. In a survey of US soldiers deployed to Iraq or Afghanistan over a 6 month period, 20% of SSTIs were culture-positive for MRSA [26]. In a Médecins Sans Frontières acute trauma hospital in Yemen, an MDR organism was identified in 60% of infections, of which skin and soft tissue infections were the most common (24%) presentation, with 73% of SA being methicillin-resistant [27].

### 1.3. Environmental Sources

SA can survive on environmental surfaces for days to months [28,29,30] with high touch-point-areas commonly found to be contaminated [31]. Environmental surveillance of this pathogen can help to identify potential hotspots in specific military training locations or deployment environments for pathogen transmission and infection to defence military uniformed members. This can inform evidence-based public health and clinical education, along with directed and modified cleaning protocols as preventative measures.

An additional reservoir of MSSA/MRSA strains for consideration of exposure to uniformed military members is local fauna throughout training locations. MRSA has been identified in Australian companion animals [32], livestock [33] and healthy wild macropods (kangaroos, wallabies, and similar) [34]. Kangaroos and other native fauna are frequently seen in military training areas throughout Australia. There are concerns amongst ADF environmental health officers that animal faeces around ranges, obstacle courses, and field environments may be transmitting SA to military members.

This study aims to investigate the following research questions and hypotheses: (1) What evidence-based solutions can be used to address a recurring unusual occurrence of SSTIs locally within ADF sites? We hypothesise that heightened personal hygiene practises in communal living settings will reduce transmission and exposure of SSTI-causing bacteria. (2) Do clinically diagnosed SSTIs result from a specific environmental or carriage strain? We will use antibiotic susceptibility testing and genomic analysis to compare isolated strains and identify transmission routes and sources. (3) Are the isolated *Staphylococcus* spp. strains endemic to Australia or imported? What virulent and resistant characteristics do they have? We hypothesise that, in a population that often travels locally and internationally, we will find a variety of local and international *Staphylococcus* spp. strains. We will characterise these strains for virulence and drug resistance profiles, and track them epidemiologically through data sharing with Australian National databases, as well as the partner nation United States multi-resistant organism repository and surveillance network of the Walter Reed Army Institute of Research (MRSN-WRAIR), Maryland.

## 2. Methodology

This study involved 2 training cohorts from an army trade-specific training site. Recruits were surveyed at the beginning and end of a 16-week training period. At both the start and end of the 16-week training period, the following were collected: nasal, axilla, and groin swabs, questionnaire data, and swabs sampling live-in accommodations, towels, linens, mattresses, gyms, obstacle courses, and designated range training areas.

### 2.1. Retrospective Review of SSTI Rates

The prevalence of SSTIs from the training site reported through the on-site health centre was collated from health system data for the 6 years prior to the study, during 2015–2021. Each clinical encounter in the health system requires a diagnosis code for data tracking purposes. As there is no definitive code for skin and soft tissue infections (SSTIs) in the health centre reporting system, a search was conducted for presentations coded as boil, MRSA, cellulitis, carbuncle, folliculitis, impetigo, SSTI, *Staphylococcal* infection, abscesses, and furuncle. A repeat clinical presentation coded under the same diagnosis code within 4 weeks of the initial presentation will be counted as one presentation in these data.

### 2.2. Participant Recruitment

Two cohorts of 48 recruits each were invited to participate, of which 51 participants met the criteria and agreed to participate. Participants gave written informed consent for the project, which was approved by the Ethics Committee of the Department of Defence and Veterans’ Affairs Human Research Ethics Committee (DDVA HREC). Inclusion criteria required trainees to be assigned to the selected site and timeframe, and to provide informed consent.

### 2.3. Sample Size and Statistical Tests

All analyses were performed using SPSS (Version 14.0, SPSS Inc., Chicago, IL, USA). Group descriptive data were reported. The results for group baseline characteristics are expressed as mean ± standard deviation. Pre- and post-training time point comparisons were performed using analysis of variance (ANOVA) techniques, utilising one-way ANOVAs and paired *t*-tests where appropriate. The level of statistical significance employed was *p* ≤ 0.05. Analysis of incidence rates and categorical data were determined using the Chi-square test. Correlation analysis of pooled data was undertaken using the Pearson coefficient with 2-tailed significance. Only those with correlations significant at the ≤0.05 level and ≤0.01 levels were reported. To have a 60% power to detect a difference in incidence risk of >9% over 16 weeks post-training period, a sample size of 49 is required. This was calculated based on the limited cohort number of 96 recruits that could be approached, expecting a volunteer rate of at least 50%. As this is a pilot study, a minimum of 30 participants were determined to be adequate for this purpose, assuming an underlying minimum of 15% SA carriers in the population.

### 2.4. Collection of Samples

Culture specimens were obtained at enrolment from the anterior nares and the right and left axilla and groin through self-collected swabs in order to assess colonisation.

To effectively identify the surfaces most frequently contaminated with bacteria like methicillin-sensitive SA (MSSA) or MRSA and allow for molecular typing, an existing clinical site sampling protocol was adapted for this survey [35,36]. Environmental samples from field training site soil material and water were collected, and in order to cover as many locations as possible within one site, samples were collected from three different triangulated locations and pooled. Specific environmental samples for individual participants were identified through their completed questionnaire. Samples were obtained from participants’ assigned bed linens, bath towels, and laundry and shower facilities. All samples were systematically collected from commonly touched surfaces in accordance with validated protocols [37,38].

### 2.5. Collection of Data from Questionnaires and Health Records

All enrolled participants subsequently completed a survey detailing their demographics, history of SSTIs or relevant pre-existing dermatologic conditions, training site living quarters, daily routines during training, recent history of antibiotic use, and recent health care facility exposures. Through this survey tool, we incorporated co-variates on age, gender, previously diagnosed SA carriage, current use of antibiotics and antimicrobial creams, self-reported SSTIs, and self-reported cuts and wounds, as well as personal practices when on field phase (i.e., sharing of toiletries and shavers, wound management, hygiene, sanitation, and rations). All questionnaire data were collated and compared within collections and between collections. Week 1 and Week 16 response data were used to identify differences and any behavioural indicators that may have affected rates of skin and soft tissue infections within the study cohort.

Health records were reviewed for all participants for the time period of the study. Any SSTIs diagnosed, antibiotics treatment given, and wound swabs taken were noted.

### 2.6. Culture for Isolation and Identification

Both swabs and material samples were brought back from the field to the lab site for processing within 48 h. Swabs were inoculated directly onto media and material samples were rinsed or suspended in sterile saline and centrifuged before inoculation of pelleted sample onto media. Thermo Scientific-Oxoid Brilliance selective agar was the chromogenic medium used for the detection and enumeration of MSSA and MRSA [39]. Columbia Horse Blood agar was used as a reference; other bacteria were isolated from the specimen including other Gram-positive bacteria. Gram staining was used to further screen samples. All suspected *Staphylococcus* spp. bacterial colonies isolated from culture and gram stains were confirmed with commercial latex agglutination kits and sent for MALDI-TOF analysis and antibiotic susceptibility testing (Melbourne Pathology, Victoria, Australia) to confirm identification to species level and associated antibiograms of isolates. All confirmed MRSA isolates were then sent for genomic sequencing (MRSN-WRAIR, Silver Spring, MD, USA).

## 3. Results

### 3.1. Baseline Findings and Retrospective SSTI Rates

In Week 1, a total of 51 participants were recruited from two separate cohorts, with participant demographics detailed in Table 1. Participants were mostly male (96%) and aged between 17 and 25 years (71%), with an average age of 23 years. Questionnaire data showed none had reported an existing skin infection or were using oral or topical antibiotics. One had previously been diagnosed with *Staphylococcus* nasal carriage and four participants had current skin wounds.

An average of 138 clinically coded SSTI presentations occurred annually at the training centre over the previous 6-year period.

Human sampling (nares, axilla, and groin swabs) and bacteria isolation demonstrated that 19% of participants had SA as normal flora on their body, without infection, with one groin sample isolating MRSA (2%). Additionally, 83% of environmental samples (220 separate samples collected) were found with *Staphylococcus* spp., of which 36% were SA, found in the indoor gym, outdoor gym, obstacle course, and accommodation (Table 2), including two (1%) MRSA samples collected from the outdoor gym.

### 3.2. Findings for 16-Week Period

**Figure 1:**
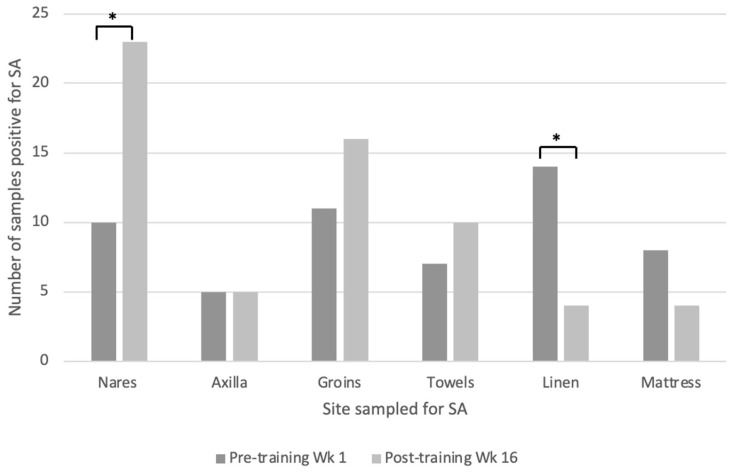
Comparing pre- and post-training samples of sites with *Staphylococcus aureus* (SA) present (* denotes significant difference with *p*-value ˂ 0.05).

Bacteria isolation from human swab sampling demonstrated a significant increase in SA nasal carriage from 19% (n = 10) to 49% (n = 23) (*p* = 0.002) over the 16-week training period, indicating transmission to participants who were not previously carriers of the bacteria (Figure 1). MRSA was identified in one groin swab, in a different participant than in the initial week, maintaining an MRSA carriage rate of 2%. Environmental samples growing SA were isolated in boxing room surfaces, accommodations, training ranges, and the obstacle course. There was no MRSA isolated from environmental samples in Week 16. There was a significant decrease in bacteria isolated on bed linen from 27% (n = 14) to 8% (n = 4) (*p* = 0.018) at the end of 16 weeks (Figure 1). It was noted that a steam cleaning of linen and mattresses by base contractors had been performed the day prior to sample collection. Within the accommodation and gym, floors and surfaces were reportedly cleaned weekly by contracted cleaning services. Soldiers were required to wash and dry their own linen and towels during their own time, and this was not enforced.

A total of three participants were lost to follow-up as they left the course for medical reasons unrelated to SSTIs. For the remaining 48 participants, questionnaire data are reported in Table 1. Skin integrity breakdown was reported in 77% of participants (n = 37), with most described as cuts, blisters, bites, rashes and bumps. Additionally, 12 of these 37 participants (32%) sought medical attention, and the remaining 25 reported self-managing their skin issues through cleaning, covering, or ignoring them. Up to 10% of participants reflected a poor understanding regarding antibacterial knowledge, listing soap, deodorants, and cold and flu tablets as antibacterials. When questioned on their opinion on how to reduce the risk of SSTIs during the course, 75% suggested altering the curriculum to include teaching basic wound management to trainees and improving the fit and functionality of personal protective equipment (body armour, knee pads, elbow pads, and gloves). The most common method for wound management during the course (when disclosed) was to clean but not cover the wound.

### 3.3. SSTIs That Developed During the 16 Weeks

Eight (16%) of study participants were diagnosed with an SSTI at the health centre during the study. All eight were treated with antibiotics. Diagnoses received included cellulitis (n = 2), folliculitis (n = 2), an infected laceration, an infected burn, an infected sinus, and a nail infection. Two participants were required to present their infection to the local emergency department. Swabs were taken for three of these participants’ SSTIs, of which two grew SA. Wound swab samples have been sent for genomic sequencing and will be subsequently investigated further when complete.

### 3.4. Bacteria Characterisation

SA bacteria were predominantly isolated from human swab samples (195 isolates) and compared to environmental samples (158 isolates). Of the 14 different *Staphylococcus* species isolated from Week 1 and 16 samples, the five of human pathogenic interest were *S. aureus*, *S. sciuri*, *S. lugdunensis*, *S. haemolyticus*, and *S. saprophyticus.* Culture-based antibiograms of all isolated SA were compared and they suggested matching patterns of SA found in Week 16 in the obstacle course, training range, and accommodation bed linen to that of initial human carriage in Week 1. Similar results were seen between human carriage in Week 1 and human carriage in Week 16.

Genomic sequencing data and accompanying antibiotic susceptibility testing are not presented in these results due to ongoing analysis.

## 4. Discussion

Comprehensive clinical and molecular epidemiology of *Staphylococcus* spp. colonisation and SSTIs in the ADF military population is important for informing preventative strategies specific to this population. It is recognised that training settings provide crowded conditions, shared equipment, and limited opportunities for personal hygiene that facilitate colonisation. The development of various wound infections during training exercises may greatly inhibit the ability of the soldier to progress through the required training continuum and can even result in medical discharge (dependent upon the severity and possible complications).

The rate of nasal carriage of SA at course initiation (baseline) was 19% (n = 10) compared to the Australian general population rate of 25–30% [40,41]. But this increased at the end of just 16 weeks to 49% (n = 23). The MRSA carriage rate of 2% was similar to that of the Australian civilian population [41]. Culture-based antibiograms of the isolated SA suggested potential transmission of carriage between humans over the 16 weeks of training together.

Military recruits’ general understanding of wound management, infection, and antibiotics could be improved. Almost 10% of participants referenced their deodorant as an antibacterial. Additionally, 77% (n = 37) of participants experienced a breakdown in skin integrity and the most common method for wound management during the course (when disclosed) was to clean but not cover the wound. The feedback from participants via the questionnaire was a desire to teach trainees how to better manage wounds in a field environment to prevent infection and to improve the fit and functionality of personal protective equipment (body armour, knee pads, elbow pads, and gloves).

Almost 16% (n = 8) of participants were clinically diagnosed with an SSTI and received antibiotic treatment. This demonstrated that even in a training environment with reasonable access to washing, laundry facilities, and medical care, SSTIs can present a significant morbidity burden.

Baseline accommodation environmental data demonstrated that SA persisted in the accommodation area despite contractor cleaning prior to course commencement. Accommodation cleaning was shown to be significantly effective in reducing persisting SA but not eliminating it from the accommodation environment. Persisting environmental SA increases the risk of exposure to pathogens and the development of SSTIs amongst arriving recruits. Other accommodation cleaning procedures, schedules, and products can be reviewed to improve the effectiveness of removing SA in between courses and keeping environmental colonisation levels low during a course. There were changes in the environmental levels of SA over the 16 weeks; this was due to the preliminary Week 1 report being released early to the site and staff. The staff at the accommodation acted on the report and performed targeted cleaning, which was evident in the reduction in the presence of isolated SA in sampling during Week 16.

The global emergence and spread of CA-MRSA have previously been well documented (A6); Australia has been no exception (A7) with ST93-IV ‘Queensland CA-MRSA’ noted as the predominant clone in Australia. Initial sequencing data demonstrate a variety of different sequence types with some potential transmission links involving specifically ST-8, ST-15, and ST-93. Whilst human-to-human transmission is suspected to be the primary source of increased nasal carriage amongst participants, environmental-to-human transmission is also being considered but no clear link was demonstrated. Whole-genome sequencing of wound swabs from SSTIs, which is currently being performed, will allow for a clear conclusion on the origin of pathogenic SA strains. Acknowledging that removing SA from the environment can be difficult and costly, particularly for an unclear benefit to soldiers, researchers aim to further investigate transmission links between person, environment, and local fauna that co-inhabit the training area.

## 5. Conclusions

The significant increase in nasal carriage of *Staphylococcus aureus* (SA) observed throughout the course suggests human-to-human transmission over the 16-week period. Confirming a link between SSTIs developed and SA in the environment or human carriage requires genome sequencing, we cannot make conclusions at this time without genomic sequencing data, which is pending. The 16% of participants diagnosed and treated for an SSTI during the 16-week period demonstrates that the morbidity of SSTIs is a burden on the training continuum at this training site and an investigation was warranted.

As a result of our findings, preventative strategies have been recommended to the training site, including targeted education in personal hygiene practices and wound management, as well as a review and enhancement of cleaning protocols. These measures aim to minimise the risk of SSTIs among military trainees and better prepare them for challenges both in training and on the battlefield.

Furthermore, this study serves as a foundation for ongoing efforts to improve cleaning and disinfection protocols across other training establishments and high-risk environments within the Australian Defence Force (ADF). By bolstering our understanding of transmission pathways and implementing proactive measures, we strive to equip our future soldiers with the knowledge and tools necessary to mitigate SSTIs effectively. In summary, the insights gained from this study underscore the importance of comprehensive preventative strategies in mitigating the burden of SSTIs in military settings, ultimately enhancing the health, readiness, and resilience of our armed forces.

## Figures and Tables

**Table 1:** Questionnaire data obtained at Week 1 and Week 16.

Week 1	
Participants	51 of 96 consented
Gender ratio Male/Female/Unanswered	49 (96%):1 (2%):1 (2%)
Age range 18–25/26–35/Unanswered	36 (71%):13 (25%):1 (2%)
Number with an existing SSTI	0
Number currently on antibiotics	0
Number previously diagnosed with SA nasal carriage	1 (2%)
Number currently using an antibacterial ointment or cream	0
**Week 16**	
Number of participants on food rations	46 (96%)
Number who developed cuts, rash, bumps, or wounds on their skin	37 (77%)
Common locations of developed cuts and wounds	Legs/knees (*n* = 16), hands (*n* = 15), arms/elbows (*n* = 14), feet (*n* = 12), face (*n* = 7), chest (*n* = 5), back (*n* = 4), groin (*n* = 1)
Number who sought medical attention if they had wounds	12/37 (32%)
Number who self-managed and did not seek medical attention	25/37 (68%)
Number who recalled sharing personal items (e.g., shaver, toothbrush, towel, clothes, hat, etc.) with other trainees	7 (15%)

**Table 2:** Outcome of environmental sampling locations that indicated the presence of SA and the study week the samples were collected.

Area	Location	Study Week
Indoor gym	Cardio equipment	1
	Weight plates	1
	Long bars	1
	Boxing room mats and gear	1 and 16
Accommodation	Common rooms	1
	Showers	1
	Laundry	1
	Towels	1 and 16
	Bed linen	1 and 16
	Mattresses	1 and 16
Outdoor gym	Weight plates	1
Range	Soil	1 and 16
Obstacle course	Leopard crawl gravel	1 and 16
	Leopard crawl sand	1 and 16

## Data Availability

The data presented in this study are available upon request from the corresponding author (the data are not publicly available due to privacy restrictions).
